# Supplementary material for: Hydroxyethylcellulose-Based Hydrogels Containing Liposomes Functionalized with Cell-Penetrating Peptides for Nasal Delivery of Insulin in the Treatment of Diabetes
Source: Pharmaceutics. 2022 Nov 17;14(11):2492. doi: 10.3390/pharmaceutics14112492 (PMC9699037; doi:10.3390/pharmaceutics14112492)
Supplement: Supplementary file 1 [file pharmaceutics-14-02492-s001.zip › pharmaceutics-1992024-supplementary.pdf]

Supplementary Information

Materials and Methods

Syringeability test

The syringeability test of hydrogels was adapted from the methodology described by Nunes and collaborators [1], using the TA-XTplus Texture Analyzer (Stable Micro Systems®). To avoid the presence of air bubbles, the formulations were carefully put into 1 mL plastic syringe (BD Plastipak™) to a height of 30 mm. The syringe was placed vertically under the probe, which was lowered to the initial contact with the plunger. The syringe plunger was moved over a distance of 30 mm, in a compression mode at a constant speed of 2 mm/s. The work required for the formulation to travel 30 mm through the syringe was determined from the area under the force-distance plot recorded during plunger compression. All measurements were carried out at 25 ºC and the results for each formulation were obtained using the mean values of six replicates.

Assessment of the mucoadhesive strength

Preparation of the mucosa models

Two mucosa models were used: porcine nasal mucosa and mucin disks. With a ring-press with a 123 mm diameter, the discs were produced by direct compression of 250 mg of powdered mucin moistened with 50 µL of mucin dispersion 8% (w / w) [2]. The discs of mucin were then horizontally connected to the lower end of the cylindrical probe (diameter 12.3 mm) with 3M double-sided adhesive tape. The porcine nasal mucosa was isolated from domestic pigs, according to the methodology adapted from Hägerström and Edsman [3]. The mucosa removal employed forceps and surgical scissors and it was kept in an ice bath and immersed in saline solution until the moment of analysis. The porcine nasal mucosa fragments were adhered to the analytical probe by a rubber ring.

Evaluation of the mucoadhesive strength

The mucoadhesive strength of formulations was evaluated by measuring the maximum strength and work required to detach the formulations from a model membrane using the TA-XTplus Texture Analyzer (Stable Micro Systems®). Before starting the experiment, the mucosa models (mucin disc and porcine nasal mucosa) already adhered to the analytical probe were immersed for 30 s in artificial nasal mucus (ANM) and the excess was removed with absorbent paper. The composition of the ANM used was 7.45 mg mL^{-1} of NaCl, 8% (w/v) of mucin type II from porcine stomach (Sigma, St. Louis, USA), 0.32 mg mL^{-1} of $CaCl_2.2H_2O$ and 1.29 mg mL^{-1} of KCl, pH adjusted to 6,0 with 0.1 M NaOH a solution [4-5]. The formulations were previously packed into shallow cylindrical vessels, placed under the analytical probe and kept immersed in a water bath at 32 ± 0.5 ºC and temperature 37 ± 0.5 ºC. After reaching the two temperatures described, the mucin disc and the porcine nasal mucosa were placed in contact with the surface of the formulations and immediately afterward a downward force of 0.1 N was applied for 30 s to ensure contact between the porcine nasal mucosa and mucin disc with the formulations. The analytical probe was

then moved upwards at a constant speed of 1.0 mm/s and the mucoadhesive strength of each formulation was determined from the resulting force-time plot and at least five replicates were carried out [6].

Results and Discussion

The study of syringeability is an important test to evaluate during the development of a delivery system that will be maintained in a specific location, since it must have adequate fluidity to facilitate application in the desired locations of the mucosa [1]. The values obtained with the syringeability test can be seen in **Table S1.** The syringeability work value of the samples was similar to each other and did not show significant difference (p value> 0.05), with values >24 N.mm, showing no change in this parameter after the incorporation of the liposomes. Additionally, the values found suggest the ease of application of the samples, in view of the lesser required force [7].

Table S1. Syringeability of the formulations at 25 °C.

**Formulations**	**Syringeability (N.mm)**
**HI**	24.83 ± 0.89
**HLI**	24.36 ± 0.52
**HLI_{TAT}**	24.67 ± 0.29
**HLI_{PNT}**	24.93 ± 0.32

The main challenge associated with the nasal administration of drugs is the permanence of the formulation in the nasal mucosa, since the drug is rapidly eliminated from the nasal cavity by a very active mucociliary clearance mechanism. Therefore, an ideal nasal drug delivery system should maintain the formulation in the nasal cavity for a prolonged period of time and thus increase its absorption and its therapeutic effect [5,8]. Thus, measurements of mucoadhesive strength were performed on two model membranes: mucin discs and porcine nasal mucosa. All formulations were tested in both models and at temperatures of 32 ± 0.5 °C and 37 ± 0.5 °C had mucoadhesive strength >0.05 N (**Figure S1**), similar to that found by the study of Basu & Bandyopadhyay [9] which proved the mucoadhesivity of gels prepared with HPMC and Carbopol 934 for nasal application.

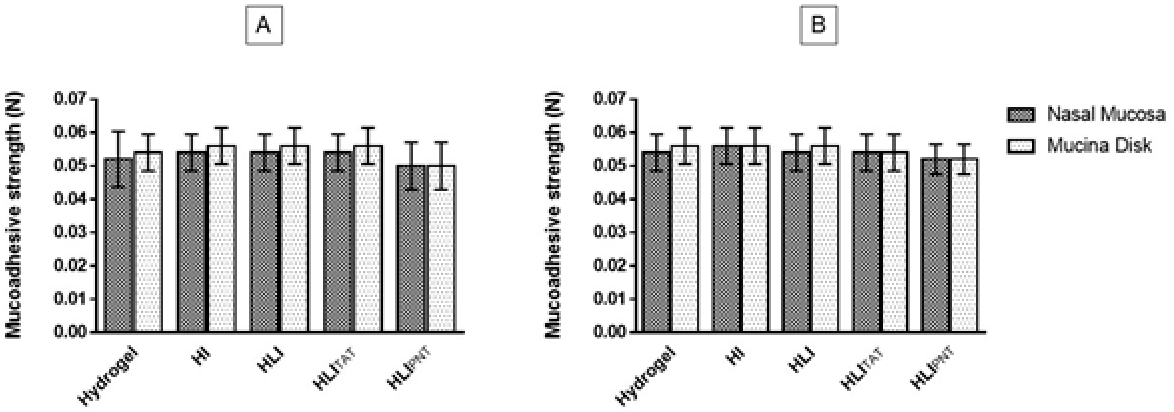

Figure S1. Comparison between mucoadhesive strength at 32 ± 0.5 °C (a) and 37 ± 0.5 °C and (b) in the formulations, using porcine nasal mucosa and mucin disc as a model membrane (n = 5). HEC-based hydrogel; HEC-based hydrogel containing insulin (HI); HEC-based hydrogel containing insulin-loaded liposomes (HLI); HEC-based hydrogel containing insulin-loaded liposomes functionalized with TAT (HLI_{TAT}) and HEC-based hydrogel containing insulin-loaded liposomes functionalized with PNT (HLI_{PNT}).

Table S2. Mathematical models and determination coefficients (R^2).

Model	Insulin Solution	HI	HLI	HLI_{TAT}	HLI_{PNT}
Baker–Londsdale	R^2=0.6949<br>k= 0.0438	R^2=0.6961<br>k= 0.0409	R^2=0.0000<br>k= 0.0084	R^2=0.1677<br>k= 0.0033	R^2=0.0367<br>k= 0.0032
Korsmeyer-Peppas	R^2 =0.7877<br>n =0.2244	R^2 =0.7263<br>n =0.1761	R^2 =0.7979<br>n =0.1716	R^2 =0.7954<br>n =0.1967	R^2 =0.7611<br>n =0.1863
Hixon–Crowell	R^2 =0.2010<br>k =61.7685	R^2 =0.0000<br>k = 59.5583	R^2 =0.0000<br>k = 38.5740	R^2 =0.0000<br>k = 25.6058	R^2 =0.0000<br>k = 26.0210
Higuchi	R^2 =0.2680<br>k = 0.0703	R^2 =0.0000<br>k = 0.0652	R^2 =0.0000<br>k = 0.0237	R^2 =0.0000<br>k = 0.0120	R^2=0.0000<br>k = 0.0118
Hopfenberg	R^2 =0.9721<br>k = 0.3169<br>n =3.000	R^2 = -0.55<br>k = 0.05<br>n =3.0000	R^2 = -1.6484<br>k = 0.0161<br>n =4.5000	R^2 = -1.4218<br>k =0.0104<br>n =3.0000	R^2= -1.6540<br>k = 0.0102<br>n =3.0000
Firt Order	R^2 =0.9667<br>k = 1.1437	R^2 =0.9348<br>k = 1.1737	R^2 =0.0000<br>k = 0.0984	R^2 =0.0000<br>k = 0.0426	R^2 =0.0000<br>k = 0.0416
Weibull	R^2 = 0.9987	R^2 = 0.9998	R^2 = 0.9992	R^2 = 0.9985	R^2= 0.9979

	k = 104.506	k = 91.0103	k = 58.7619	k =41.5194	k = 40.7869
	b = 0.7157	*b* = 1.1295	*b* = 0.9691	*b* = 0.8999	*b* = 0.9824

Note: k (mg/mL.h^{-1})

Referências

1. Nunes, K. M.;Teixeira, C. C.; Kaminski, R. C.; Sarmento, V. H.; Couto, R. O.; Pulcinelli, S. H.; Freitas, O. The monoglyceride content affects the self-assembly behavior, rheological properties, syringeability, and mucoadhesion of in situ–gelling liquid crystalline phase. *Journal of Pharmaceutical Sciences*. **2016**, 105, 2355-2364. https://doi.org/10.1016/j.xphs.2016.05.005.
2. Bruschi, M. L.; Jones, D. S.; Panzeri, H.; Gremião, M. P. D.; Freitas, O.; Lara, E. H. G. Semisolid systems containing propolis for the treatment of periodontal disease: in vitro release kinetics, syringeability, rheological, textural, and mucoadhesive properties. *Journal of Pharmaceutical Sciences***. 2007**, 96, 2074–2089.
3. Hägerström, H.; Edsman, K. Interpretation of Mucoadhesive Properties of Polymer. *Journal of Pharmacy and Pharmacology* **2001**, *53*, 1589–1599.
4. Callens, C.; Ceulemans, J.; Ludwig, A.; Foreman, P.; Remon, J. P. Rheological Study on Mucoadhesivity of Some Nasal Powder Formulations. *European Journal of Pharmaceutics and Biopharmaceutics* **2003**, *55*, 323–328. https://doi.org/10.1016/S0939-6411(03)00024-9.
5. Carvalho, F. C.; Campos, M. L.; Peccinini, R. G.; Gremião, M. P. D. Nasal Administration of Liquid Crystal Precursor Mucoadhesive Vehicle as an Alternative Antiretroviral Therapy. *European Journal of Pharmaceutics and Biopharmaceutics* **2013**, *84*, 219–227. https://doi.org/10.1016/j.ejpb.2012.11.021.
6. Gupta, P. N.; Pattani, A.; Curran, R. M.; Kett, V. L.; Andrews, G. P.; Morrow, R. J.; Woolfson, A. D.; Malcolm, R. K. Development of Liposome Gel Based Formulations for Intravaginal Delivery of the Recombinant HIV-1 Envelope Protein CN54gp140. *European Journal of Pharmaceutical Sciences* **2012**, *46*, 315–322. https://doi.org/10.1016/j.ejps.2012.02.003.
7. El, Kechai, N.; Bochot, A.; Huang, N.; Nguyen, Y.; Ferrary, E.; Agnely, F. Effect of Liposomes on Rheological and Syringeability Properties of Hyaluronic Acid Hydrogels Intended for Local Injection of Drugs. *International Journal of Pharmaceutics* **2015**, *487*, 187–196. https://doi.org/10.1016/j.ijpharm.2015.04.019.
8. Karavasili, C.; Fatouros, D. G. Smart Materials: In Situ Gel-Forming Systems for Nasal Delivery. *Drug Discovery Today* **2016**, *21*, 157–166. https://doi.org/10.1016/j.drudis.2015.10.016.
9. Basu, S.; Bandyopadhyay, A. K. Development and characterization of mucoadhesive in situ nasal gel of midazolam prepared with Ficus carica mucilage. *AAPS PharmSciTech*. **2010**, 11, 1223-1231. https://doi.org/10.1208/s12249-010-9477-x
