# Supplementary figures and images for: Hydroxyethylcellulose-Based Hydrogels Containing Liposomes Functionalized with Cell-Penetrating Peptides for Nasal Delivery of Insulin in the Treatment of Diabetes
Source: Pharmaceutics. 2022 Nov 17;14(11):2492. doi: 10.3390/pharmaceutics14112492 (PMC9699037; doi:10.3390/pharmaceutics14112492)

Uncropped, original image of western blot for phospho-[Ser-473]-AKT

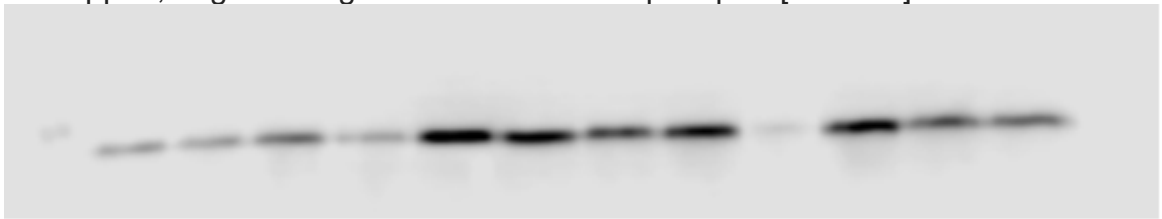

Membrane, Ponceau S staining

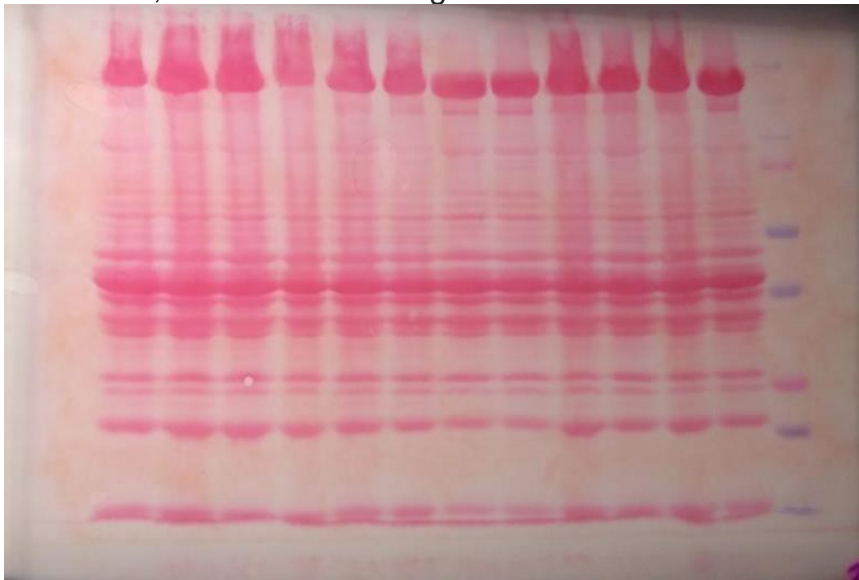

Supplement: Supplementary file 1 [file pharmaceutics-14-02492-s001.zip › WB file.pdf]
